# Outcome of the Masquelet Technique for Complex Bilateral Distal Femoral Bone Defects

**DOI:** 10.7759/cureus.38503

**Published:** 2023-05-03

**Authors:** Ziad A Aljaafri, Abdullah Alzahrani, Ali Alshehri, Ahmed AlHussain, Faisal Alzahrani, Khalid Alsheikh

**Affiliations:** 1 College of Medicine, King Saud Bin Abdulaziz University for Health Sciences, Riyadh, SAU; 2 Department of Orthopedic Surgery, King Abdullah International Medical Research Center, Riyadh, SAU; 3 Department of Orthopedic Surgery, King Abdulaziz Medical City, Riyadh, SAU; 4 Department of Orthopaedic Surgery, King Saud Bin Abdulaziz University for Health Sciences College of Medicine, Riyadh, SAU

**Keywords:** bilateral distal femur, mva (motor vehicle accident), case report, intraarticular fractures, bone defect, masquelet technique

## Abstract

Bone defects are severe conditions caused by various etiologies, including trauma, tumor resection, or chronic osteomyelitis. Different surgical interventions can be utilized to manage such cases, including autologous graft or allograft implantation, distraction osteogenesis, acute shortening, amputation, or the induced membrane technique. Herein, the case of a 39-year-old woman with complex bilateral distal femoral fractures with intra-articular extension is presented. The fractures were accompanied by a significant metaphyseal bone defect, which was managed successfully using the induced membrane Masquelet technique. The patient fully healed despite residual knee joint contractures that did not inhibit her mobility. In conclusion, the Masquelet technique successfully manages complex bone defects and restores functionality even in bilateral simultaneous open bone defects.

## Introduction

Bone defects are severe conditions caused by various etiologies, such as trauma, tumor removal, or chronic osteomyelitis. A series of cellular, humoral, and mechanical processes are involved in bone healing and remodeling, restoring bone integrity [1]. Surgical interventions should be performed in most cases, and specific precautions must be taken when long-bone segmental defects of the upper or lower limbs are present [2].

The surgical methods for treating long-bone defects include autologous bone graft or allograft implantation, distraction osteogenesis, acute shortening, vascularized fibular graft transfer, amputation, and the induced membrane technique [1-3]. Since its inception in the 1980s, the Masquelet technique has been used to treat nonunion fractures and bone defects. The first stage involves debridement, fracture stabilization, bone cement insertion, and tension-free wound closure. The second stage involves cutting open the induced membrane, which takes six to eight weeks or more, depending on the anatomical location of the defect. Bone grafting is then conducted [4-9].

This case report demonstrates the surgical approach and outcomes of the Masquelet technique in a morbidly obese patient with bilateral open intra-articular distal femoral fractures with a metaphyseal bone defect sized 6 cm and 8 cm in the right and left distal femurs, respectively. The presence of post-traumatic bilateral femoral bone defects with a similar anatomical profile makes this case report of substantial value for other surgeons facing this rare occurrence. This paper will outline the management and special challenges that could arise.

## Case presentation

A 39-year-old woman patient with a history of hypertension and morbid obesity was transferred to our hospital 20 days after a rollover motor vehicle accident. She was initially transferred to a peripheral health care center, and her bilateral knee wounds were sutured in their emergency department. She was then transferred to the regional hospital in her area, where she was kept on skin traction, and no surgical intervention was performed.

**Figure 1:**
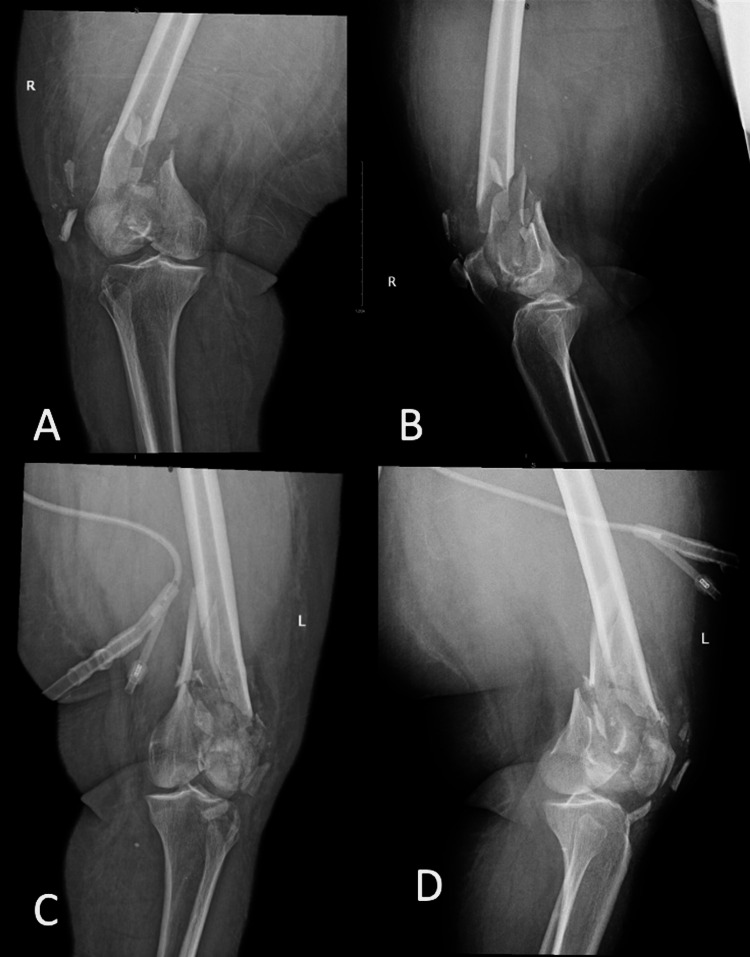
(A) Anteroposterior and (B) lateral radiographs of the right distal femur. (C) Anteroposterior and (D) lateral radiographs of the left distal femur.

The patient was alert and oriented with unremarkable primary and secondary survey findings, except for bilateral knee pain and wounds. She was noted to be morbidly obese, with a BMI of 44 kg/m2. During an examination of the lower limbs, the right lower limb showed a sutured mid-knee wound sized approximately 4 cm with no discharge, no signs of infection, good ankle range of motion, and intact distal neurovascular status. A mid-knee wound sized approximately 3 cm was also sutured in the left lower limb, with purulent discharge, good ankle range of motion, and intact distal neurovascular status. Initial radiographic studies of the right and left distal femurs are shown in Figure 1. The radiographs showed bilateral intra-articular distal femoral fractures with metaphyseal comminution and shortening.

**Figure 2:**
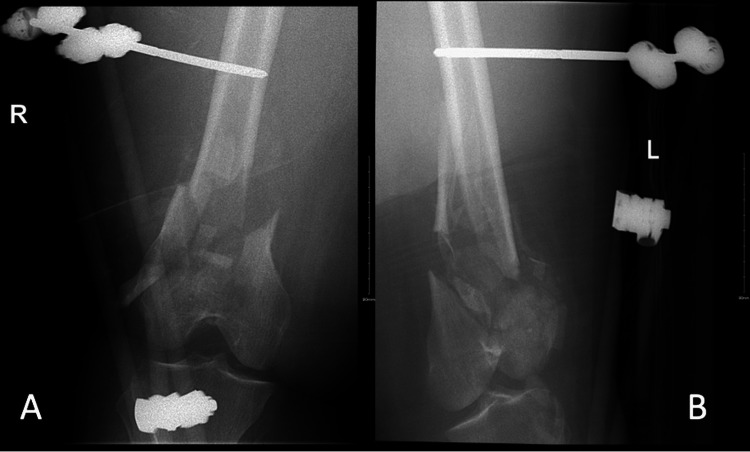
(A) Anteroposterior radiograph of the right distal femur. (B) Anteroposterior radiograph of the left distal femur.

The initial plan was to assess inflammatory markers and perform a bilateral knee CT. The knee CT scans showed bilateral open intra-articular distal femoral fractures with metaphyseal comminution associated with a large collection bilaterally at the distal third medial thigh, which was most likely an intramuscular hematoma. The patient underwent bilateral distal femoral irrigation and debridement (I&D) and spanning external fixation (Figure 2).

The anesthesia team put the patient on patient-controlled analgesia (PCA), during which the patient experienced mild pain but no side effects. Thereafter, intraoperative cultures (fluid, tissue, and bone) were conducted. The infectious diseases team was consulted, and cefazolin treatment was continued. The cultures were negative on day three post-I&D and external fixation. Pin site dressing and patient care were performed in accordance with our protocol, and cefazolin treatment was continued until the final culture results were obtained.

The surgical wound was clean and dry on day seven post-I&D and external fixation, and a bone scan or single-photon emission computerized tomography (SPECT) showed no evidence of an infectious process. On day 10, she underwent another I&D and was kept on enoxaparin and cefazolin, as well as external fixation.

**Figure 3:**
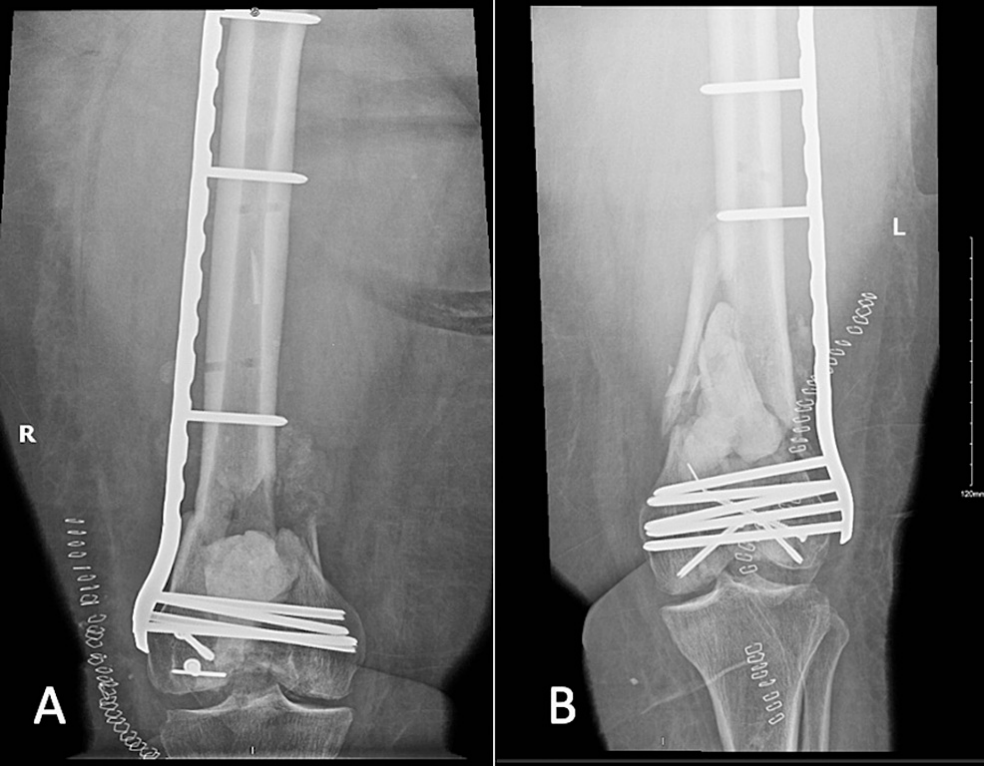
(A) Anteroposterior radiograph of the right distal femur. (B) Anteroposterior radiograph of the left distal femur.

Five days after the second I&D, the patient underwent another I&D of the bilateral distal femurs with open reduction, internal fixation, and placement of cement mixed with vancomycin and gentamicin (the first stage of the Masquelet technique). The procedure was successfully performed with no complications, and the patient was put on patient-controlled analgesia (PCA) morphine for pain control (Figure 3).

Postoperatively, the patient required a blood transfusion of two units of packed red blood cells. The patient showed negative cultures but a high clinical suspicion of osteomyelitis. Thus, the infectious diseases team was consulted for antibiotic management. They planned to put the patient on vancomycin and tazocin for 21 days.

Twenty days later, the patient was noted to have a limited range of motion of the knees (0°-20° bilaterally). After patient counseling, she underwent manipulation of the bilateral knees under anesthesia. Her treatment regimen was changed to tazocin and linezolid when her left knee wound produced some discharge during manipulation for a possible surgical site infection. The patient was discharged after a 14-day antibiotic course in good condition and with healed knee wounds.

**Figure 4:**
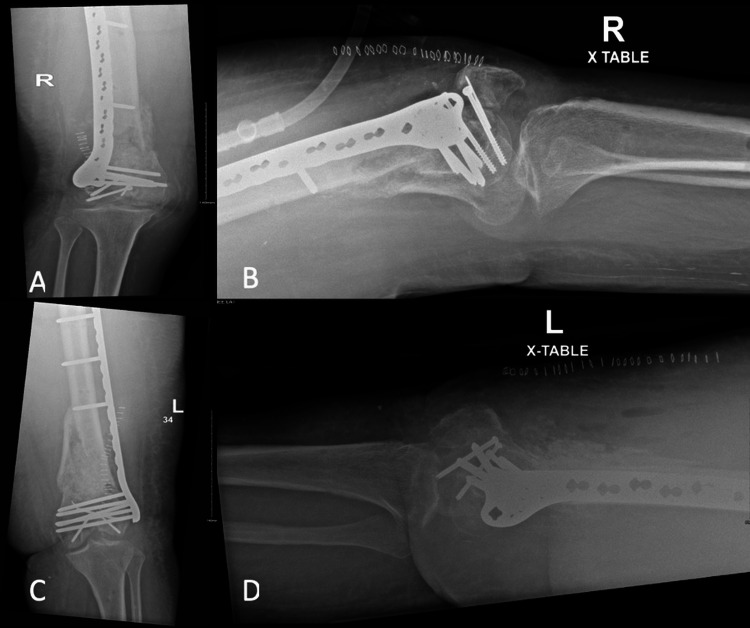
(A) Anteroposterior and (B) lateral radiographs of the right distal femur. (C) Anteroposterior and (D) lateral radiographs of the left distal femur.

After 12 weeks, the patient was admitted for the second stage of the Masquelet technique but was found to have left lower limb swelling. The patient underwent venous Doppler ultrasound, which showed extensive deep vein thrombosis. The hematology team was consulted, and enoxaparin and an IVC filter were recommended. After optimization, the patient underwent surgery. In particular, she underwent the removal of bilateral distal femoral cement, the placement of a bone graft, and another manipulation of the bilateral knees (Figure 4). The surgery was successfully performed, and the patient was put on vancomycin, tazocin, and enoxaparin. The antibiotic management was based on clinical suspicion, despite no tissue evidence of infection. The IVC filter was removed a week later, and the patient was stable. After completing the antibiotic course, the patient was discharged home in good condition.

**Figure 5:**
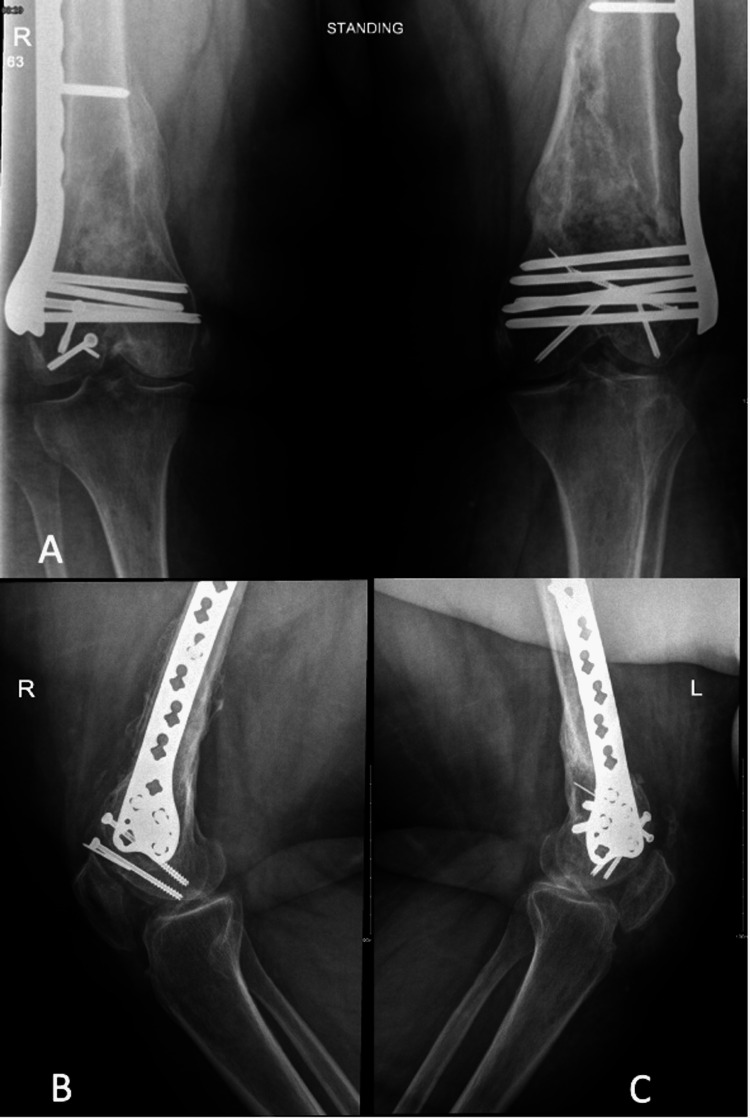
(A) Anteroposterior radiograph of the bilateral distal femur. (B) Lateral radiograph of the right distal femur; (C) Lateral radiograph of the left distal femur.

The patient was followed up in our orthopedic trauma clinic at two, six, and 12 weeks post-second-stage Masquelet technique, during which she showed neither radiographic signs of graft resorption nor clinical or laboratory signs of infection. At 20 weeks, she was able to bear weight on her bilateral lower limbs with no pain, and the radiographs showed complete bone bridging, indicating full union. However, she had limited flexion at 10° bilaterally but full extension. She electively underwent bilateral arthroscopic-assisted release of knee joint contractures. One year later, she had full extension and 60°-70° flexion on her bilateral knees and was satisfied with her outcomes (Figure 5).

## Discussion

The coexistence of bilateral open distal femoral fractures and substantial bone defects is uncommon. The goal of management is to preserve mobility and achieve the healing of bone defects. It is also essential to save the limb from complications that might arise, including osteomyelitis and instability. Ultimately, it is imperative to restore patients' independence and functionality. Intra-articular distal femoral fractures with meta-diaphyseal bone defects can be managed using several methods, including external fixation, open reduction and internal fixation, intramedullary nailing, or bone cementing and grafting [10-15].

A recent case series showed a similar case of a patient who presented with a unilateral open distal femoral fracture after a motor vehicle accident. A bone defect sized approximately 6 cm was noted, which was successfully managed using the two-stage Masquelet technique. Patient functionality was restored, and the union was achieved within one year of serial follow-ups [16]. Similarly, the bone defects in our patient were approximately 6 cm on the right and 8 cm on the left. However, the bilaterality and simultaneous occurrence of the defects, along with the state of both injuries being open fractures, delayed interventions owing to logistical reasons. All these circumstances in the patient with a challenging body habitus make this case of special interest. Moreover, during serial follow-ups, our patient reached clinical and radiographic union, defined as complete bone bridging on three or more cortices, within five months of the second-stage surgery.

In our institution, a suspected or established infection is typically managed in accordance with our protocol. After cultures and laboratory workups are performed, medical management is provided by the infectious diseases team. Such management is aided by a follow-up on inflammatory markers and sensitivity test results, and recommendations on the duration and type of antibiotic treatment are followed.

In the present case, definitive fixation after the two-stage Masquelet technique was achieved using a distal femoral anatomical variable-angle locking plate and inter-fragmentary screws for articular fragments, a standard and valid construct for such fractures [17].

Joint contracture is a known complication of distal femoral fractures. Patient education regarding common complications and their possible management is crucial. Our patient was noted to have joint contractures multiple times throughout the follow-ups, for which physiotherapy sessions were not helpful enough. After the second stage of the Masquelet technique, our patient electively underwent bilateral arthroscopic-assisted release of knee joint contractures and was satisfied with her outcomes. Various studies have also reported joint contracture [18] and explained stiffness to be likely to occur in the presence of intra-articular fractures, external fixators, and prolonged immobilization. These factors were all present in our patient.

The Masquelet technique has recently received much attention for its promising outcomes, especially its high union rate and low complication rate following bone defects [19]. Moreover, as shown in our case, this approach successfully restores the mobility and functionality of patients.

## Conclusions

Herein, the Masquelet technique was proven to be successful in treating complex bilateral distal femoral bone defects. Patient functionality was regained within five months of the second stage of the Masquelet technique. Joint contracture was noticed throughout the follow-ups, with limited improvement following physiotherapy sessions, for which the patient underwent arthroscopic-assisted release. The patient was satisfied with the results. Earlier management in a specialized center could have reduced the pre-and postoperative morbidity rates and yielded better outcomes within a shorter time frame. A well-prepared trauma center with specialized surgeons with good expertise could help deal with complex cases and improve overall outcomes.
